# Crystal structure of *N*-[(1*S*,2*S*)-2-amino­cyclo­hex­yl]-2,4,6-tri­methyl­benzene­sulfonamide

**DOI:** 10.1107/S205698901502191X

**Published:** 2015-11-21

**Authors:** Felix N. Ngassa, Shannon M. Biros, Richard J. Staples

**Affiliations:** a Department of Chemistry, Grand Valley State University, 1 Campus Dr., Allendale, MI 49401, USA; b Center for Crystallographic Research, Department of Chemistry, Michigan State University, 578 S. Shaw Lane, East Lansing, MI 48824, USA

**Keywords:** crystal structure, sulfonamide, hydrogen bond, chiral compound

## Abstract

The title compound, C_15_H_24_N_2_O_2_S, was synthesized *via* a substitution reaction between the enanti­opure (1*S*,2*S*)-(+)-1,2-di­amino­cyclo­hexane and 2,4,6-tri­methyl­benzene-1-sulfonyl chloride. The cyclo­hexyl and phenyl substituents are oriented *gauche* around the sulfonamide S—N bond. In the crystal, mol­ecules are linked *via* N—H⋯N hydrogen bonds, forming chains propagating along [100].

## Abstract 2

In the crystal structure of the title compound, the sulfonamide N—H group forms an inter­molecular hydrogen bond to the amine N atom.

## Chemical context   

Many sulfonamides have been reported as anti­cancer, anti-inflammatory, and anti­viral agents (Navia, 2000 ▸; Yan *et al.*, 2006 ▸; Palakurthy & Mandal, 2011 ▸). The use of sulfonamides as catalysts in asymmetric synthesis has also been reported (Lao *et al.*, 2009 ▸; Feng *et al.*, 2010 ▸; Jin *et al.*, 2010 ▸). Through explicit hydrogen-bonding inter­actions with specific functional groups, the electrophilicity and stereoselectivity of a given substrate is enhanced.

Conjugate addition reactions of aldehydes and ketones to nitro­alkenes, catalyzed by chiral primary amines, have been reported (Huang & Jacobsen, 2006 ▸; Rabalakos & Wulff, 2008 ▸; Lao *et al.*, 2009 ▸; Sun *et al.*, 2012 ▸; Zhou *et al.*, 2014 ▸; Ruiz-Olalla *et al.*, 2015 ▸; Yang *et al.*, 2015 ▸). The catalytic activity of chiral primary amine organocatalysts with particular emphasis on the role of the N—H acidity and hydrogen bonding has also been investigated (Lao *et al.*, 2009 ▸). Although the N—H acidity and hydrogen-bonding modes could have an effect on the catalytic activity of the organocatalysts, the nature of the substrate and reaction conditions could be more important. Asymmetric conjugate addition reactions of aldehydes to nitro­alkenes have also been reported as a convenient synthesis of γ-amino acids (Horne & Gellman, 2008 ▸; Wiesner *et al.*, 2008 ▸; Chi *et al.*, 2008 ▸).
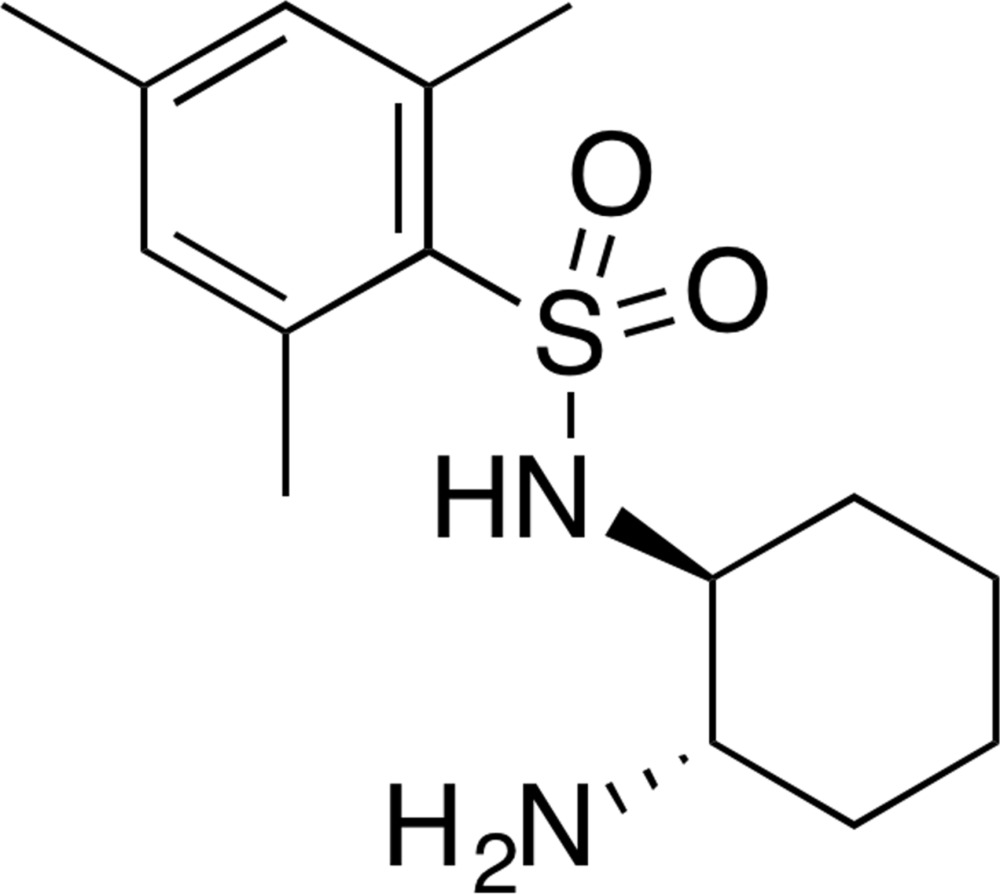


In line with our research inter­est in the synthesis of heterogeneous foldamers (Hayen *et al.*, 2004 ▸), we synthesized the title compound as a chiral organocatalyst for conjugate addition. This conjugate addition was then applied for the synthesis of γ-amino acids, which have been shown to be inter­esting foldamer building blocks (Horne & Gellman, 2008 ▸). Therefore, as the title compound is of inter­est in our ongoing effort on foldamer design and synthesis, we report here on the synthesis and crystal structure of this chiral sulfonamide.

## Structural commentary   

**Figure 1:**
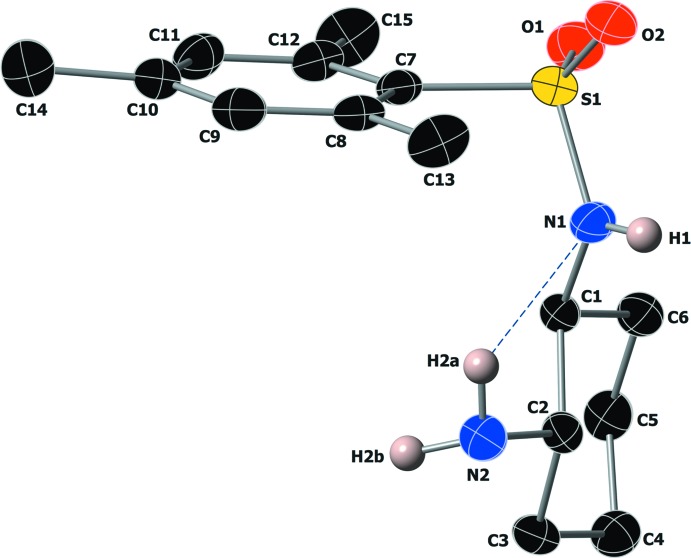
The asymmetric part of the unit cell along with the atom-numbering scheme and displacement ellipsoids drawn at the 50% probability level. An intra­molecular N—H⋯N inter­action is shown with a blue dashed line. Only N—H hydrogens are shown for clarity.

The asymmetric part of the unit cell is shown in Fig. 1 ▸ along with the atom-numbering scheme. The absolute stereochemistry of this chiral sulfonamide was confirmed by a Flack parameter of 0.00 (2) (Parsons *et al.*, 2013 ▸). The cyclo­hexyl (C1–C6) and benzene (C7–C12) substituents are oriented *gauche* around the sulfonamide S—N bond, with a C1—N1—S1—C7 torsion angle of 70.4 (2)°. A weak intra­molecular inter­action is present between the amine H2*A* atom and the *sp* ^2^-hybridized sulfonamide N1 atom (Table 1 ▸).

As described in the *Database survey* section below, the structure of a racemic crystal of this compound has been reported (FAVHEP; Balsells, *et al.*, 1998 ▸). In this crystal, there are two crystallographically unique mol­ecules of the sulfonamide compound in the asymmetric unit. Here, the cyclo­hexyl and benzene substituents are oriented gauche around the S—N bond with torsion angles of 86.8 (8) and 69.1 (7)°. While we expected that there would be an intra­molecular hydrogen bond in this crystal, in the model deposited in the CSD there are no intra­molecular hydrogen bonds present between the amine N—H group and the sulfonamide N atom.

## Supra­molecular features   

**Figure 2:**
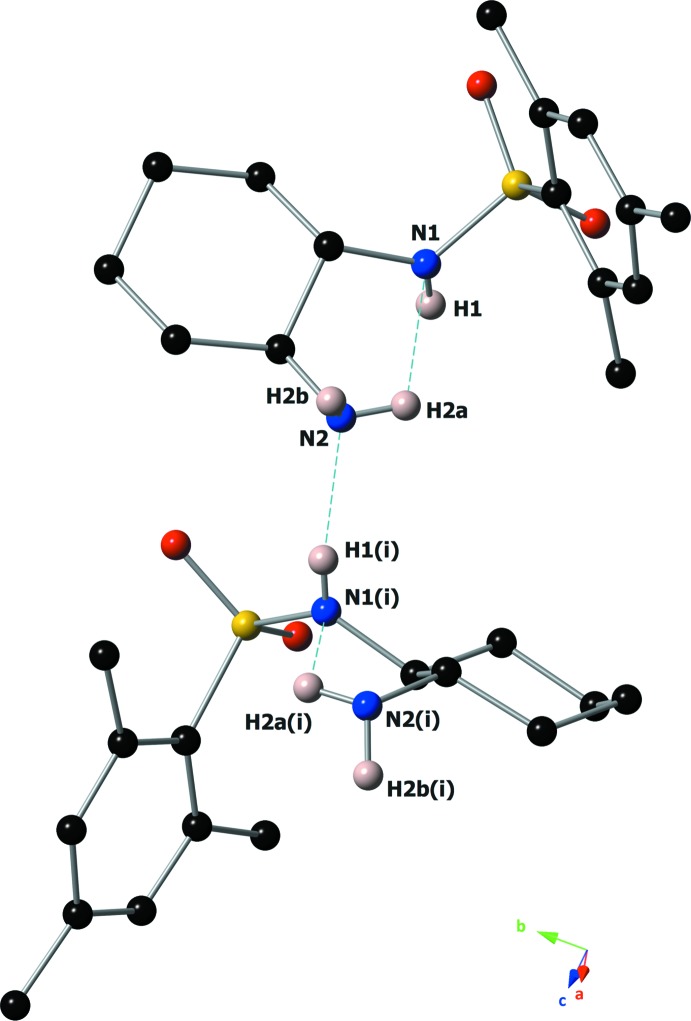
Intra- and inter­molecular hydrogen-bonding inter­actions present in the crystal. Hydrogen bonds are drawn as blue dashed lines. Only N—H hydrogens are shown for clarity. [Symmetry code: (i) *x* − 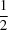, −*y* + 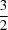, −*z* + 1.]

Mol­ecules of the title compound are held together in the solid state by inter­molecular hydrogen-bonding inter­actions between the donor sulfonamide N1—H1 and the acceptor amine N2 atoms (Table 1 ▸ and Fig. 2 ▸). These hydrogen bonds arrange mol­ecules into supra­molecular chains that are oriented along the [100] axis (Fig. 2 ▸). Weaker N2—H2*B*⋯O1(1 + *x*, *y*, *z*) inter­actions with an H2*B*⋯O1(1 + *x*, *y*, *z*) distance of 2.72 Å between the donor amine N2—H2*B* and the acceptor sulfonamide O1 atoms can also be noticed within this chain.

As for the racemic crystal FAVHEP, in the model deposited in the CSD there is one inter­molecular hydrogen bond present between a donor sulfonamide N1—H1 and a nearby amine acceptor N atom [*D*⋯H = 0.860 (7) Å; H⋯*A* = 2.160 (8) Å; *D*⋯*A* = 3.011 (8) Å; *D*—H⋯*A* = 169.9 (5)°].

## Database survey   

The Cambridge Structural Database (CSD, Version 5.36, May 2015; Groom & Allen, 2014 ▸) contains 35 sulfonamides bearing a mesitylene group on the S atom. Of these, there are four structures where the substituent bonded to the sulfonamide N atom is an aliphatic six-membered ring. In structures RAWMAF (Hou *et al.*, 2012 ▸) and ZIQPAS (Wu *et al.*, 2014 ▸), the amino­cyclo­hexane substituent is part of a larger fused-ring system. Inter­estingly, there are two structures with 1,2-di­amino­cyclo­hexane rings as the amide substituent. In structure OTOPAP (Schwarz *et al.*, 2010 ▸), both amines of the *trans*-1,2-di­amino­cyclo­hexane ring are bonded to a mesitylsulfonamide group. Structure FAVHEP (Balsells *et al.*, 1998 ▸) is the same as the title compound, but is present as a racemic mixture that crystallized in the space group *P* 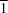.

## Synthesis and crystallization   

To a stirred solution of (1*S*,2*S*)-(+)-1,2-di­amino­cyclo­hexane (0.77 g, 6.74 mmol) in 5 ml of CH_2_Cl_2_ at 273 K was added a solution of 2,4,6-tri­methyl­benzene-1-sulfonyl chloride (0.44 g, 2.01 mmol) in 5 ml CH_2_Cl_2_. After the addition was complete (20 min), the mixture was allowed to warm to room temperature and stirred overnight. The reaction mixture was washed with H_2_O (3 × 25 ml) and the aqueous layer was back-extracted with CH_2_Cl_2_ (20 ml). The combined organic extracts were dried over Na_2_SO_4_ and the solvent was removed under reduced pressure. The residue was purified by column chromatography over silica gel (CH_2_Cl_2_/EtOAc 1:1 *v*/*v*) to afford a pale-yellow–white solid (yield: 0.46 g, 78%). Part of the purified product was redissolved in CH_2_Cl_2_ and after slow evaporation for several days, white large chunky crystals (stained yellow) were formed that were suitable for analysis by X-ray diffraction (m.p. 406–407 K).

## Refinement   

Crystal data, data collection and structure refinement details are summarized in Table 2 ▸. The positions of all non-polar H atoms were calculated geometrically and refined to ride on their parent atoms, with *U*_iso_(H) = 1.2*U*_eq_(C) for methine, methyl­ene and aryl groups, and *U*_iso_(H) = 1.5*U*_eq_(C) for methyl groups. H atoms bonded directly to N atoms (H1, H2*A* and H2*B*) were located in difference-Fourier maps and refined isotropically.

## Supplementary Material

Crystal structure: contains datablock(s) I. DOI: 10.1107/S205698901502191X/gk2646sup1.cif

Structure factors: contains datablock(s) I. DOI: 10.1107/S205698901502191X/gk2646Isup2.hkl

Supporting information file. DOI: 10.1107/S205698901502191X/gk2646Isup3.cml

CCDC reference: 1437453

Additional supporting information:  crystallographic information; 3D view; checkCIF report

## Figures and Tables

**Table 1:** Hydrogen-bond geometry (Å, °)

*D*—H⋯*A*	*D*—H	H⋯*A*	*D*⋯*A*	*D*—H⋯*A*
N2—H2*A*⋯N1	0.89 (3)	2.43 (3)	2.877 (3)	111 (2)
N1—H1⋯N2^i^	0.79 (3)	2.14 (3)	2.921 (3)	170 (3)

Symmetry code: (i) 
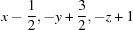
.

**Table 2:** Experimental details

Crystal data	
Chemical formula	C_15_H_24_N_2_O_2_S
*M*_r_	296.42
Crystal system, space group	Orthorhombic, *P*2_1_2_1_2_1_
Temperature (K)	173
*a*, *b*, *c* (Å)	6.5215 (4), 10.0202 (6), 23.3660 (15)
*V* (Å^3^)	1526.89 (16)
*Z*	4
Radiation type	Mo *K*α
μ (mm^−1^)	0.22
Crystal size (mm)	0.37 × 0.20 × 0.15

Data collection	
Diffractometer	Bruker APEXII CCD
Absorption correction	Multi-scan (*SADABS*; Bruker, 2014 ▸)
*T*_min_, *T*_max_	0.706, 0.745
No. of measured, independent and observed [*I* > 2σ(*I*)] reflections	25587, 2799, 2667
*R*_int_	0.034
(sin θ/λ)_max_ (Å^−1^)	0.602

Refinement	
*R*[*F* ^2^ > 2σ(*F* ^2^)], *wR*(*F* ^2^), *S*	0.029, 0.071, 1.06
No. of reflections	2799
No. of parameters	196
H-atom treatment	H atoms treated by a mixture of independent and constrained refinement
Δρ_max_, Δρ_min_ (e Å^−3^)	0.19, −0.21
Absolute structure	Flack parameter *x* determined using 1098 quotients [(*I* ^+^)−(*I* ^−^)]/[(*I* ^+^)+(*I* ^−^)] (Parsons *et al.*, 2013 ▸)
Absolute structure parameter	0.00 (2)

Computer programs: *APEX2* ad *SAINT* (Bruker, 2013 ▸), *SHELXS97* (Sheldrick, 2008 ▸), *SHELXL2014* (Sheldrick, 2015 ▸), *OLEX2* (Dolomanov *et al.*, 2009 ▸; Bourhis *et al.*, 2015 ▸) and *CrystalMaker* (Palmer, 2007 ▸).

## supplementary crystallographic information

### Crystal data

**Table:**

C_15_H_24_N_2_O_2_S	*D*_x_ = 1.289 Mg m^−^^3^
*M**_r_* = 296.42	Mo *K*α radiation, λ = 0.71073 Å
Orthorhombic, *P*2_1_2_1_2_1_	Cell parameters from 9968 reflections
*a* = 6.5215 (4) Å	θ = 2.2–25.3°
*b* = 10.0202 (6) Å	µ = 0.22 mm^−^^1^
*c* = 23.3660 (15) Å	*T* = 173 K
*V* = 1526.89 (16) Å^3^	Block, colourless
*Z* = 4	0.37 × 0.20 × 0.15 mm
*F*(000) = 640	

### Data collection

**Table:**

Bruker APEXII CCD diffractometer	2799 independent reflections
Radiation source: sealed tube	2667 reflections with *I* > 2σ(*I*)
Graphite monochromator	*R*_int_ = 0.034
Detector resolution: 8 pixels mm^-1^	θ_max_ = 25.4°, θ_min_ = 1.7°
φ and ω scans	*h* = −7→7
Absorption correction: multi-scan (*SADABS*; Bruker, 2014)	*k* = −12→12
*T*_min_ = 0.706, *T*_max_ = 0.745	*l* = −28→28
25587 measured reflections	

### Refinement

**Table:**

Refinement on *F*^2^	Hydrogen site location: mixed
Least-squares matrix: full	H atoms treated by a mixture of independent and constrained refinement
*R*[*F*^2^ > 2σ(*F*^2^)] = 0.029	*w* = 1/[σ^2^(*F*_o_^2^) + (0.0313*P*)^2^ + 0.5049*P*] where *P* = (*F*_o_^2^ + 2*F*_c_^2^)/3
*wR*(*F*^2^) = 0.071	(Δ/σ)_max_ < 0.001
*S* = 1.06	Δρ_max_ = 0.19 e Å^−^^3^
2799 reflections	Δρ_min_ = −0.21 e Å^−^^3^
196 parameters	Absolute structure: Flack parameter *x* determined using 1098 quotients [(*I*^+^)-(*I*^-^)]/[(*I*^+^)+(*I*^-^)] (Parsons *et al.*, 2013)
0 restraints	Absolute structure parameter: 0.00 (2)

### Special details

**Table:**

Geometry. All e.s.d.'s (except the e.s.d. in the dihedral angle between two l.s. planes) are estimated using the full covariance matrix. The cell e.s.d.'s are taken into account individually in the estimation of e.s.d.'s in distances, angles and torsion angles; correlations between e.s.d.'s in cell parameters are only used when they are defined by crystal symmetry. An approximate (isotropic) treatment of cell e.s.d.'s is used for estimating e.s.d.'s involving l.s. planes.

### Fractional atomic coordinates and isotropic or equivalent isotropic displacement parameters (Å^2^)

**Table:**

	*x*	*y*	*z*	*U*_iso_*/*U*_eq_	
S1	0.39425 (9)	0.81200 (6)	0.62892 (2)	0.03018 (16)	
O1	0.2464 (3)	0.72704 (19)	0.65596 (8)	0.0412 (4)	
O2	0.3238 (3)	0.93797 (17)	0.60697 (8)	0.0408 (5)	
N1	0.5015 (3)	0.7361 (2)	0.57616 (9)	0.0297 (5)	
N2	0.9140 (3)	0.6769 (2)	0.54061 (8)	0.0287 (4)	
C1	0.5776 (3)	0.5984 (2)	0.58084 (9)	0.0238 (5)	
H1A	0.6425	0.5872	0.6193	0.029*	
C2	0.7442 (3)	0.5816 (2)	0.53502 (9)	0.0234 (5)	
H2	0.6780	0.5986	0.4971	0.028*	
C3	0.8271 (3)	0.4398 (2)	0.53360 (10)	0.0273 (5)	
H3A	0.9052	0.4225	0.5692	0.033*	
H3B	0.9228	0.4306	0.5009	0.033*	
C4	0.6574 (4)	0.3363 (2)	0.52793 (10)	0.0308 (5)	
H4A	0.7174	0.2457	0.5300	0.037*	
H4B	0.5902	0.3460	0.4902	0.037*	
C5	0.4980 (4)	0.3528 (2)	0.57518 (11)	0.0314 (5)	
H5A	0.3864	0.2871	0.5696	0.038*	
H5B	0.5624	0.3353	0.6128	0.038*	
C6	0.4094 (4)	0.4934 (2)	0.57442 (10)	0.0300 (5)	
H6A	0.3095	0.5031	0.6061	0.036*	
H6B	0.3356	0.5080	0.5379	0.036*	
C7	0.5988 (4)	0.8413 (2)	0.67770 (9)	0.0252 (5)	
C8	0.7523 (4)	0.9336 (2)	0.66163 (9)	0.0270 (5)	
C9	0.9071 (4)	0.9619 (2)	0.69984 (10)	0.0321 (5)	
H9	1.0119	1.0225	0.6887	0.039*	
C10	0.9154 (4)	0.9054 (2)	0.75383 (10)	0.0340 (6)	
C11	0.7662 (4)	0.8131 (3)	0.76822 (10)	0.0346 (5)	
H11	0.7724	0.7723	0.8049	0.042*	
C12	0.6078 (4)	0.7775 (2)	0.73148 (9)	0.0291 (5)	
C13	0.7568 (5)	1.0087 (2)	0.60541 (10)	0.0384 (6)	
H13A	0.7084	0.9502	0.5746	0.058*	
H13B	0.8974	1.0373	0.5972	0.058*	
H13C	0.6675	1.0871	0.6080	0.058*	
C14	1.0799 (5)	0.9459 (3)	0.79566 (13)	0.0532 (8)	
H14A	1.2117	0.9523	0.7757	0.080*	
H14B	1.0897	0.8789	0.8261	0.080*	
H14C	1.0452	1.0327	0.8124	0.080*	
C15	0.4607 (5)	0.6726 (3)	0.75300 (12)	0.0440 (7)	
H15A	0.3236	0.7116	0.7569	0.066*	
H15B	0.5071	0.6399	0.7903	0.066*	
H15C	0.4559	0.5983	0.7258	0.066*	
H2A	0.864 (5)	0.754 (3)	0.5537 (12)	0.047 (8)*	
H1	0.484 (5)	0.768 (3)	0.5458 (13)	0.040 (8)*	
H2B	1.003 (5)	0.645 (3)	0.5683 (13)	0.045 (8)*	

### Atomic displacement parameters (Å^2^)

**Table:**

	*U*^11^	*U*^22^	*U*^33^	*U*^12^	*U*^13^	*U*^23^
S1	0.0271 (3)	0.0327 (3)	0.0307 (3)	0.0072 (3)	0.0005 (3)	−0.0065 (2)
O1	0.0275 (8)	0.0496 (11)	0.0465 (10)	−0.0037 (8)	0.0092 (8)	−0.0111 (8)
O2	0.0427 (10)	0.0387 (10)	0.0410 (10)	0.0196 (8)	−0.0057 (8)	−0.0067 (8)
N1	0.0371 (11)	0.0293 (11)	0.0228 (10)	0.0109 (9)	−0.0014 (9)	−0.0010 (9)
N2	0.0283 (10)	0.0250 (10)	0.0329 (10)	−0.0021 (9)	0.0002 (9)	−0.0004 (9)
C1	0.0239 (11)	0.0250 (11)	0.0225 (10)	0.0044 (9)	−0.0012 (9)	−0.0009 (9)
C2	0.0228 (10)	0.0238 (11)	0.0236 (10)	0.0009 (9)	0.0001 (9)	0.0016 (9)
C3	0.0261 (11)	0.0259 (12)	0.0300 (12)	0.0053 (10)	0.0036 (9)	0.0003 (9)
C4	0.0357 (13)	0.0241 (11)	0.0326 (12)	0.0008 (10)	0.0001 (10)	−0.0010 (9)
C5	0.0315 (13)	0.0313 (13)	0.0315 (12)	−0.0059 (10)	0.0014 (10)	0.0010 (10)
C6	0.0229 (11)	0.0359 (12)	0.0312 (11)	−0.0006 (11)	0.0032 (11)	−0.0033 (10)
C7	0.0278 (11)	0.0242 (10)	0.0235 (10)	0.0038 (10)	0.0040 (10)	−0.0040 (8)
C8	0.0327 (12)	0.0217 (11)	0.0267 (11)	0.0030 (10)	0.0081 (10)	−0.0020 (9)
C9	0.0291 (12)	0.0290 (12)	0.0382 (13)	−0.0015 (11)	0.0069 (12)	−0.0045 (10)
C10	0.0319 (13)	0.0361 (13)	0.0340 (13)	0.0076 (11)	−0.0008 (11)	−0.0102 (10)
C11	0.0465 (14)	0.0342 (12)	0.0232 (11)	0.0075 (13)	0.0014 (11)	0.0010 (10)
C12	0.0367 (12)	0.0247 (11)	0.0259 (11)	0.0018 (11)	0.0062 (11)	−0.0010 (9)
C13	0.0536 (16)	0.0285 (12)	0.0331 (13)	−0.0021 (12)	0.0098 (13)	0.0058 (11)
C14	0.0455 (18)	0.0629 (19)	0.0512 (17)	0.0037 (16)	−0.0124 (16)	−0.0140 (15)
C15	0.0569 (18)	0.0351 (14)	0.0401 (14)	−0.0079 (13)	0.0094 (13)	0.0066 (12)

### Geometric parameters (Å, º)

**Table:**

S1—O1	1.4330 (19)	C6—H6A	0.9900
S1—O2	1.4379 (18)	C6—H6B	0.9900
S1—N1	1.609 (2)	C7—C8	1.414 (3)
S1—C7	1.779 (2)	C7—C12	1.411 (3)
N1—C1	1.470 (3)	C8—C9	1.377 (3)
N1—H1	0.79 (3)	C8—C13	1.514 (3)
N2—C2	1.469 (3)	C9—H9	0.9500
N2—H2A	0.89 (3)	C9—C10	1.384 (3)
N2—H2B	0.93 (3)	C10—C11	1.384 (4)
C1—H1A	1.0000	C10—C14	1.507 (4)
C1—C2	1.535 (3)	C11—H11	0.9500
C1—C6	1.527 (3)	C11—C12	1.390 (4)
C2—H2	1.0000	C12—C15	1.509 (3)
C2—C3	1.521 (3)	C13—H13A	0.9800
C3—H3A	0.9900	C13—H13B	0.9800
C3—H3B	0.9900	C13—H13C	0.9800
C3—C4	1.522 (3)	C14—H14A	0.9800
C4—H4A	0.9900	C14—H14B	0.9800
C4—H4B	0.9900	C14—H14C	0.9800
C4—C5	1.525 (3)	C15—H15A	0.9800
C5—H5A	0.9900	C15—H15B	0.9800
C5—H5B	0.9900	C15—H15C	0.9800
C5—C6	1.523 (3)		
			
O1—S1—O2	117.62 (12)	C1—C6—H6A	109.4
O1—S1—N1	110.46 (11)	C1—C6—H6B	109.4
O1—S1—C7	108.68 (11)	C5—C6—C1	111.33 (19)
O2—S1—N1	106.31 (11)	C5—C6—H6A	109.4
O2—S1—C7	108.86 (11)	C5—C6—H6B	109.4
N1—S1—C7	104.06 (11)	H6A—C6—H6B	108.0
S1—N1—H1	116 (2)	C8—C7—S1	117.96 (16)
C1—N1—S1	122.24 (17)	C12—C7—S1	121.78 (19)
C1—N1—H1	120 (2)	C12—C7—C8	120.2 (2)
C2—N2—H2A	108 (2)	C7—C8—C13	124.7 (2)
C2—N2—H2B	108.1 (18)	C9—C8—C7	118.8 (2)
H2A—N2—H2B	107 (3)	C9—C8—C13	116.5 (2)
N1—C1—H1A	108.4	C8—C9—H9	118.8
N1—C1—C2	106.87 (18)	C8—C9—C10	122.4 (2)
N1—C1—C6	113.39 (19)	C10—C9—H9	118.8
C2—C1—H1A	108.4	C9—C10—C14	120.6 (3)
C6—C1—H1A	108.4	C11—C10—C9	117.9 (2)
C6—C1—C2	111.36 (17)	C11—C10—C14	121.5 (2)
N2—C2—C1	113.59 (18)	C10—C11—H11	118.5
N2—C2—H2	107.1	C10—C11—C12	123.0 (2)
N2—C2—C3	109.98 (18)	C12—C11—H11	118.5
C1—C2—H2	107.1	C7—C12—C15	125.9 (2)
C3—C2—C1	111.70 (18)	C11—C12—C7	117.7 (2)
C3—C2—H2	107.1	C11—C12—C15	116.5 (2)
C2—C3—H3A	109.1	C8—C13—H13A	109.5
C2—C3—H3B	109.1	C8—C13—H13B	109.5
C2—C3—C4	112.32 (19)	C8—C13—H13C	109.5
H3A—C3—H3B	107.9	H13A—C13—H13B	109.5
C4—C3—H3A	109.1	H13A—C13—H13C	109.5
C4—C3—H3B	109.1	H13B—C13—H13C	109.5
C3—C4—H4A	109.4	C10—C14—H14A	109.5
C3—C4—H4B	109.4	C10—C14—H14B	109.5
C3—C4—C5	111.02 (19)	C10—C14—H14C	109.5
H4A—C4—H4B	108.0	H14A—C14—H14B	109.5
C5—C4—H4A	109.4	H14A—C14—H14C	109.5
C5—C4—H4B	109.4	H14B—C14—H14C	109.5
C4—C5—H5A	109.5	C12—C15—H15A	109.5
C4—C5—H5B	109.5	C12—C15—H15B	109.5
H5A—C5—H5B	108.1	C12—C15—H15C	109.5
C6—C5—C4	110.51 (19)	H15A—C15—H15B	109.5
C6—C5—H5A	109.5	H15A—C15—H15C	109.5
C6—C5—H5B	109.5	H15B—C15—H15C	109.5
			
S1—N1—C1—C2	−156.04 (17)	C2—C1—C6—C5	55.2 (2)
S1—N1—C1—C6	80.9 (2)	C2—C3—C4—C5	−55.0 (3)
S1—C7—C8—C9	177.22 (17)	C3—C4—C5—C6	56.8 (3)
S1—C7—C8—C13	−0.7 (3)	C4—C5—C6—C1	−57.3 (3)
S1—C7—C12—C11	−175.90 (18)	C6—C1—C2—N2	−177.74 (19)
S1—C7—C12—C15	4.4 (3)	C6—C1—C2—C3	−52.6 (2)
O1—S1—N1—C1	−46.1 (2)	C7—S1—N1—C1	70.4 (2)
O1—S1—C7—C8	−173.93 (17)	C7—C8—C9—C10	−1.4 (3)
O1—S1—C7—C12	4.7 (2)	C8—C7—C12—C11	2.7 (3)
O2—S1—N1—C1	−174.73 (18)	C8—C7—C12—C15	−177.0 (2)
O2—S1—C7—C8	−44.7 (2)	C8—C9—C10—C11	2.8 (4)
O2—S1—C7—C12	133.97 (19)	C8—C9—C10—C14	−176.1 (2)
N1—S1—C7—C8	68.36 (19)	C9—C10—C11—C12	−1.4 (4)
N1—S1—C7—C12	−112.99 (19)	C10—C11—C12—C7	−1.3 (4)
N1—C1—C2—N2	57.9 (2)	C10—C11—C12—C15	178.4 (2)
N1—C1—C2—C3	−176.97 (18)	C12—C7—C8—C9	−1.5 (3)
N1—C1—C6—C5	175.8 (2)	C12—C7—C8—C13	−179.4 (2)
N2—C2—C3—C4	179.90 (18)	C13—C8—C9—C10	176.7 (2)
C1—C2—C3—C4	52.8 (3)	C14—C10—C11—C12	177.4 (2)

### Hydrogen-bond geometry (Å, º)

**Table:**

*D*—H···*A*	*D*—H	H···*A*	*D*···*A*	*D*—H···*A*
N2—H2*A*···N1	0.89 (3)	2.43 (3)	2.877 (3)	111 (2)
N1—H1···N2^i^	0.79 (3)	2.14 (3)	2.921 (3)	170 (3)

Symmetry code: (i) *x*−1/2, −*y*+3/2, −*z*+1.
